# MFS transporter from *Botrytis cinerea* provides tolerance to glucosinolate-breakdown products and is required for pathogenicity

**DOI:** 10.1038/s41467-019-10860-3

**Published:** 2019-06-28

**Authors:** David Vela-Corcía, Dhruv Aditya Srivastava, Avis Dafa-Berger, Neta Rotem, Omer Barda, Maggie Levy

**Affiliations:** Department of Plant Pathology and Microbiology, the Robert H. Smith Faculty of Agriculture, Food and Environment, The Hebrew University of Jerusalem, Rehovot, 76100 Israel

**Keywords:** Microbiology, Fungi, Fungal host response, Fungal pathogenesis, Plant sciences

## Abstract

Glucosinolates accumulate mainly in cruciferous plants and their hydrolysis-derived products play important roles in plant resistance against pathogens. The pathogen *Botrytis cinerea* has variable sensitivity to glucosinolates, but the mechanisms by which it responds to them are mostly unknown. Exposure of *B*. *cinerea* to glucosinolate-breakdown products induces expression of the Major Facilitator Superfamily transporter, *mfsG*, which functions in fungitoxic compound efflux. Inoculation of *B. cinerea* on wild-type *Arabidopsis thaliana* plants induces *mfsG* expression to higher levels than on glucosinolate-deficient *A. thaliana* mutants. A *B. cinerea* strain lacking functional *mfsG* transporter is deficient in efflux ability. It accumulates more isothiocyanates (ITCs) and is therefore more sensitive to this compound in vitro*;* it is also less virulent to glucosinolates-containing plants. Moreover, *mfsG* mediates ITC efflux in *Saccharomyces cerevisiae* cells, thereby conferring tolerance to ITCs in the yeast. These findings suggest that *mfsG* transporter is a virulence factor that increases tolerance to glucosinolates.

## Abstract (web summary)

Plant glucosinolates are important in defense against fungal pathogens. Here, the authors identify a major facilitator superfamily transporter protein of the pathogen *Botrytis cinerea*, *mfsG*, that plays a role in efflux and detoxification of glucosinolate-breakdown products during plant–pathogen interactions.

## Introduction

One of the key plant defense mechanisms against pathogenic microorganisms relies on the production of phytoalexins^1–3^ and phytoanticipins^4,5^, such as the amino-acid-derived metabolites camalexin and glucosinolate (GS), respectively^6^. Fungal pathogens use various pathogenicity and virulence strategies to efficiently detoxify or eliminate these phytoanticipins and phytoalexins^5,7,8^. Detoxification can be achieved by enzymatic conversion of phytoanticipins and phytoalexins in many phytopathogenic fungi, whereas elimination can be achieved by active efflux, as shown, for example, in *Botyrytis cinerea* for the phytoalexins resveratrol and eugenol^7–11^.

In several fungal species, active efflux by ATP-binding cassette (ABC) or major facilitator superfamily (MFS) transporters provides resistance to various toxic compounds, including not only secondary metabolites but also antibiotics and fungicides^12–14^. Although crucial to pathogenicity on plants, only a limited number of ABC transporters have been studied for their role in pathogenesis, for example, *Magnaporthe grisea* ABC1, ABC3 and ABC4 on rice^15–17^, *B. cinerea* atrB on grapevine and *Arabidopsis thaliana*^10,14^, *Gibberella pulicaris* ABC1 on potato^18^, and *Mycosphaerella graminicola* atr4 on wheat^19^.

It is important to note that ABC and MFS transporters are not limited to plant pathogens; their active efflux is used by many other organisms as well^20^. In the human pathogens *Candida* and *Aspergillus* spp., for example, these transporters contribute to resistance to azole fungicide, making therapy less efficient^21^. The ABC transporter AFR1 of the human pathogen *Cryptococcus neoformans* provides defense against fluconazole and acts as a virulence factor by protecting the fungi from toxic compounds produced by the host phagocytes^22^. Probably one of the best-studied bacterial ABC transporters is LmrA of *Lactobacillus lactis*, which contributes to bacterial antibiotic resistance in humans^23,24^.

We recently showed that *B. cinerea* displays variable sensitivity to GSs and their degradation products, whereas *Alternaria brassicicola*, a specialist *Brassica* pathogen, was more tolerant to GS and its hydrolysis products^25^. Although the toxicity of isothiocyanates (ITCs) and other GS-hydrolysis products to a range of fungi has been demonstrated, the mechanism of toxicity and the fungi’s ability to tolerate or detoxify GS-hydrolysis products are still largely obscure.

*B. cinerea* is a necrotrophic plant pathogen with a broad host range ( >200 plant hosts), including the agriculturally important cruciferous crops. It is the causal agent of gray mold, resulting in enormous economic losses, both during plant growth and in the postharvest phase^26,27^. *B. cinerea* control depends on repeated use of chemical fungicides, which causes rapid development of fungicide resistance^28^. Thus, understanding the mechanisms by which this pathogen detoxifies its host defense system is important for revealing basic cellular processes and crucial for the development of new strategies to control this pathogen.

Here, we report on the identification of a *B. cinerea* MFS transporter, designated *mfsG*, involved in detoxifying ITCs. This transporter was differentially expressed in the presence of different ITCs in vitro, and was upregulated in planta during interaction with wild-type *A. thaliana*. A *B. cinerea* isolate with *mfsG* deletion (*ΔmfsG*) was less able to tolerate ITCs in vitro, and therefore showed reduced virulence toward *A. thaliana*. *B. cinerea mfsG* enabled tolerance to benzyl ITC (BITC) in the heterologous *Saccharomyces cerevisiae* system, further supporting our findings that *mfsG* functions in the efflux of GS-breakdown products during plant–pathogen interactions.

## Results

### ITCs inhibit mycelial growth of *B. cinerea* in vitro

**Fig. 1:**
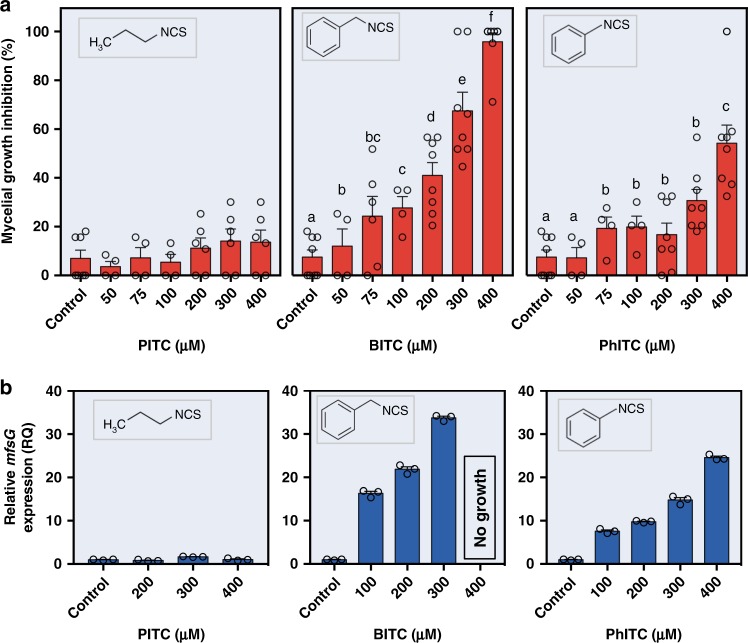
Growth inhibition of *B. cinerea* and *mfsG* expression in response to ITCs. **a** Diameter of *B. cinerea* colonies was measured 48 h post inoculation on PDA plates supplemented with different concentrations of PITC, BITC or PhITC. Growth inhibition was calculated as percentage of colony diameter of *B. cinerea* grown on PDA with no ITCs. Data are displayed as mean values (± SE), *n* > 4. Different letters above the columns indicate significant difference at *P* < 0.05, as determined by Tukey–Kramer HSD test. **b** Expression of *mfsG* 72 h after exposure to different concentrations of PITC (left), BITC (middle) or PhITC (right), as determined by qRT-PCR, relative to expression on PDA without ITCs. *n* = 3. Source data are provided as a Source Data file

To determine the inhibitory effect of the defense-related GS-breakdown products—ITCs—on *B. cinerea* (isolate B05.10), radial growth was tested in the presence of different concentrations of propyl ITC (PITC), BITC, and 2-phenethyl ITC (PhITC). Both BITC and PhITC inhibited radial growth in a dose-dependent manner (Fig. 1a), whereas the inhibitory effect of PITC was minor (Fig. 1a). *B. cinerea* was most sensitive to BITC, with complete (100%) inhibition at 400 μm. In comparison, only 70% inhibition was observed with 400 μm PhITC (Fig. 1a).

### Identification of a MFS gene whose expression is induced by ITCs

RNA-Seq analysis of the GS-breakdown product BITC revealed 16 differentially expressed MFS genes; only one of them was strongly upregulated and was designated *mfsG* (unpublished data; Supplementary Fig. 1).

### Expression of *mfsG* is induced by ITCs

Quantitative real-time PCR (qRT-PCR) analysis, using different concentrations of BITC and PhITC, revealed *mfsG* upregulation in response to BITC (Fig. 1b). The expression level of *mfsG* was dependent on BITC and PhITC concentration. Increasing concentrations of BITC increased the relative quantity of mRNA copies of *mfsG*, with a peak at 300 μm BITC. Above this concentration, *B. cinerea* was unable to grow and relative quantification of mRNA could not be determined. Corresponding to the growth-inhibition results, *mfsG* expression was lower in the presence of PITC and PhITC compared with BITC (Fig. 1b).

### *mfsG*-knockout mutants are impaired in ITC efflux and inhibited by ITCs in vitro

**Fig. 2:**
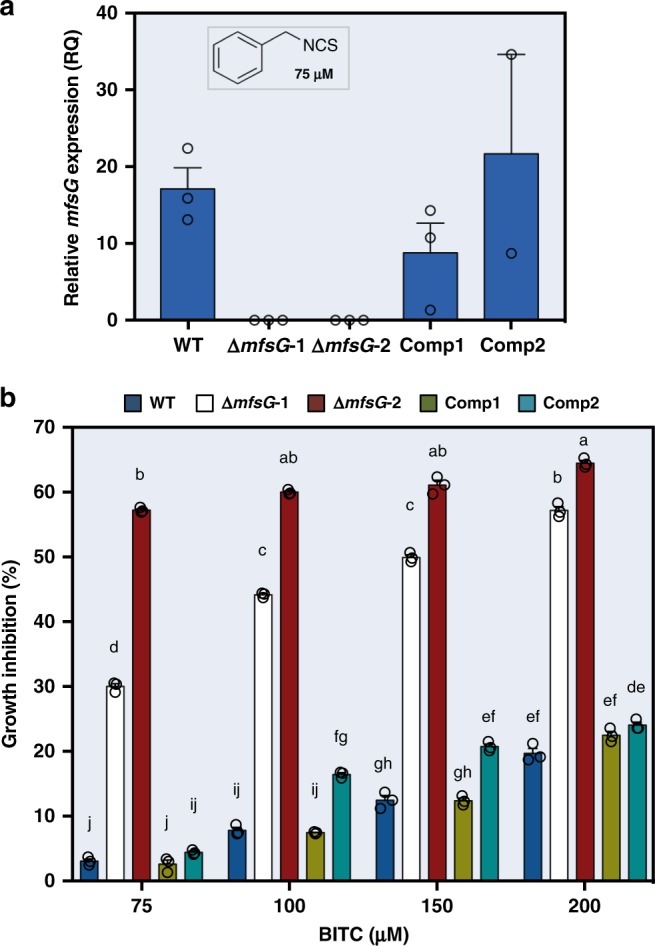
Characterization of Δ*mfsG* mutants and complemented mutants. **a** Expression analysis of mutants and complemented mutants 72 h after exposure to 75 μM BITC, *n* = 3. **b** In vitro growth inhibition 72 h after exposure to different concentrations of BITC. Growth inhibition was calculated as percentage of colony diameter of wild-type (WT) *B. cinerea* grown on PDA with no ITCs. Data are displayed as mean values (± SE). Different letters above the columns indicate significant difference at *P* < 0.05, as determined by Kruskal–Wallis test, *n* = 3. Source data are provided as a Source Data file

**Fig. 3:**
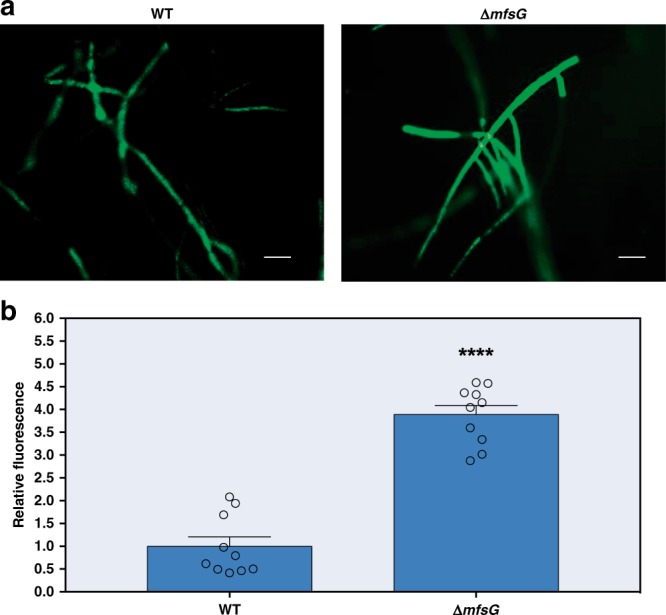
*mfsG* confers ITC efflux in *B. cinerea*. **a** Fluorescence accumulation in *ΔmfsG* relative to the wild type (WT) 4 days after exposure to 25 mg ml^−1^ FITC. **b** Quantification of fluorescence accumulation in *ΔmfsG* relative to WT hyphae. Data are displayed as mean values (± SE) of relative fluorescence. Asterisk denotes significant difference by Student’s *t* test (*P* *<* 0.001), *n* = 10. Scale bars are 20 μm. Source data are provided as a Source Data file

Knockout mutants of *mfsG* (*ΔmfsG-1 and ΔmfsG-2*) did not exhibit *mfsG* expression (Fig. 2a). As expected, these mutants’ growth was already restricted at the low BITC concentration (10 μm; Supplementary Fig. 2). After 24 h, the *ΔmfsG*s were inhibited by 65% on 200 μm BITC, whereas the wild-type strain was only inhibited by 20% (Fig. 2b). Complementation of *ΔmfsG* with the full-length *mfsG* in two strains, Comp1 and Comp2, restored *mfsG* expression (Fig. 2a) and significantly reduced growth inhibition by BITC as compared with the knockout lines, to levels comparable to the wild-type strain (Fig. 2b). Furthermore, we demonstrated the *ΔmfsG* hyphae are impaired in ITC efflux and accumulate four times more fluorescent ITC (FITC) than wild-type *B. cinerea* (Fig. 3).

### *mfsG* expression is modulated by ITC content in planta

**Fig. 4:**
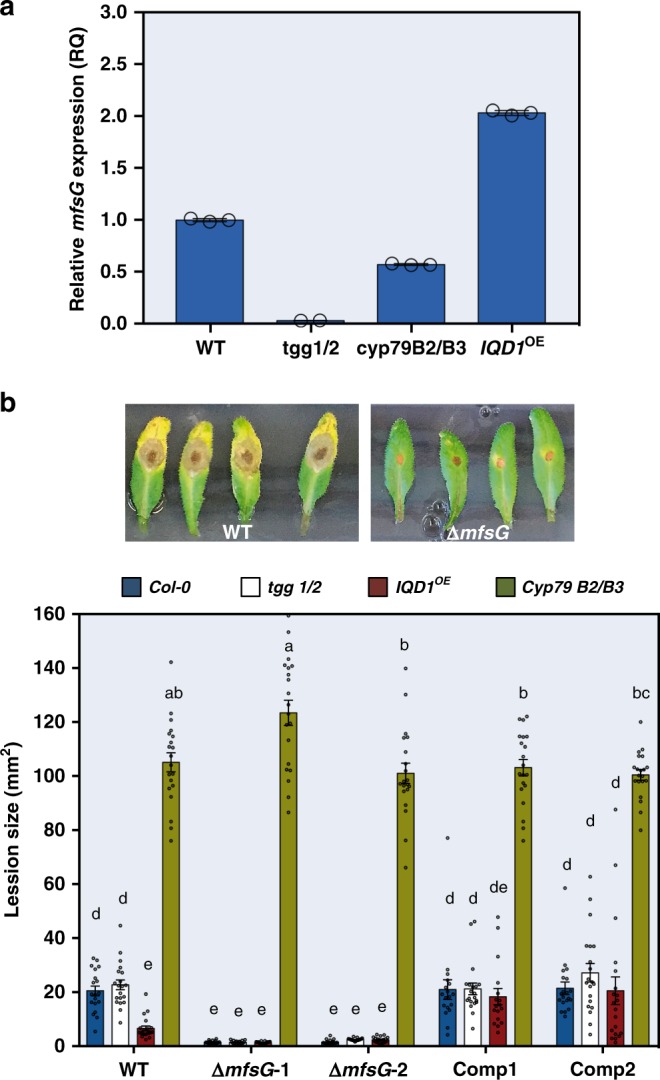
*mfsG* expression levels and pathogenicity assays on *Arabidopsis* plants. **a** *mfsG* expression analysis 48 h after inoculation of wild-type *B. cinerea* on *Arabidopsis* wild type (WT; *Col-0*), *IQD1*^OE^, *cyp79B2/B3* and *tgg1/2*, *n* = 3. **b** Pathogenicity of *B. cinerea* wild type (WT), knockout mutants (Δ*mfsG*), and complemented mutants (Comp) was evaluated on different *Arabidopsis* genotypes by measuring lesion sizes 72 h after inoculation with the different *B. cinerea* strains (representative pictures are presented for *Col-0* Arabidopsis leaves inoculated with WT B05.10 or Δ*mfsG*). Average lesion sizes of 17–20 leaves of each genotype are presented together with the standard errors for each average. Different letters above the columns indicate significant difference at *P* < 0.05, as determined by Kruskal–Wallis test. Source data are provided as a Source Data file

To study the expression levels of *mfsG in planta*, we used different *A. thaliana* genotypes with different GS levels: IQD1-overexpressing plants (*IQD1*^*OE*^) which contain double the amount of GS^29^ compared with the wild type, the double-mutant *cyp79B2/cyp79B3* (*cyp79B2/B3*) that accumulates only a quarter of the wild-type amount of GS^30^ and double-mutant *tgg1-3/tgg2-1* (*tgg1/2*) that contains wild-type GS levels but is impaired in both myrosinase hydrolytic enzymes^31^. The mutant and IQD1^OE^ plants were inoculated with *B. cinerea*, and *mfsG* expression levels were determined 48 h post inoculation as compared with wild-type plants. As expected, *mfsG* transporter was upregulated in plants containing high GS levels (*IQD1*^*OE*^), whereas it was downregulated in plants with low GS levels (*cyp79B2/B3*) and in plants that do not accumulate GS-breakdown products (*tgg1/2*) (Fig. 4a).

### *ΔmfsG* mutants are less virulent in planta

To determine the virulence of *ΔmfsG* mutants in response to GS levels in planta, we performed pathogenicity tests of wild-type *B. cinerea*, its knockout mutant and complemented mutant on the GS-overexpressing and mutant *A. thaliana* genotypes. *B. cinerea ΔmfsG* mutants were 80% less virulent on wild-type *A. thaliana* plants compared with the wild-type *B. cinerea*, as determined by measuring the lesion sizes 72 h post inoculation (Fig. 4b). The *ΔmfsG* mutants did not produce any significant lesions on plant wild-type plants, or *tgg1*/2 or *IQD1*^OE^ plants, all compose normal or high levels of GS. However, they produced lesions with sizes comparable to those produced by the *B. cinerea* wild-type strain on the double-mutant *cyp79B2/B3*, which has reduced GSs levels (Fig. 4b). Disease severity reached *B. cinerea* wild-type levels when infecting the *A. thaliana* wild type, *tgg1/2* and *IQD1*^OE^ with the *B. cinerea* complementants of *mfsG* (Fig. 4b).

### *ΔmfsG* mutants demonstrate lower frequency of double germ tubes

**Fig. 5:**
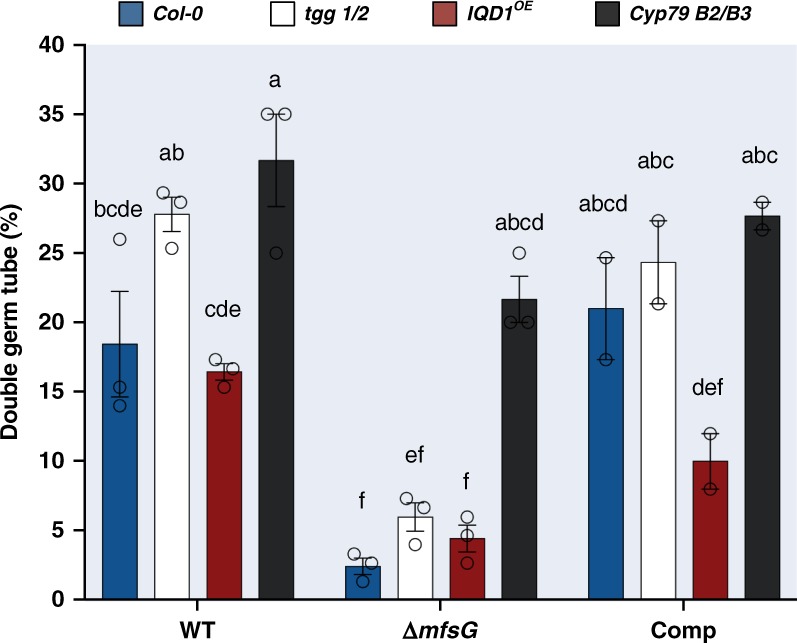
Germination test of *B. cinerea* Δ*mfsG* mutants and complemented mutants. The number of double germ tubes in germinating conidia was evaluated by bright-field microscopy 24 h after inoculation of *Arabidopsis* wild type (WT; *Col-0*), *IQD1*^OE^ and *tgg1/2* with *B. cinerea* WT, knockout mutants (Δ*mfsG*) and complemented mutants (Comp). Data are displayed as mean values (± SE). Different letters above the columns indicate significant difference at *P* < 0.05, as determined by Tukey–Kramer HSD test, *n* > 20. Source data are provided as a Source Data file

To evaluate the fitness of the wild-type, knockout mutant and complemented *B. cinerea*, the percentages of conidial germination and double germ-tube production were evaluated by microscopy. The germination rate of all strains was close to 100% with no significant differences among them. However, *ΔmfsG* mutants showed a significantly lower percentage of double germ tubes than wild-type strain on all *A. thaliana* genotypes. After mutant complementation, the number of double germ tubes was comparable to the wild type (Fig. 5). The highest number of double germ tubes was observed in *cyp79B2/B3* and *tgg1/2*, and the lowest, albeit not significantly, in *IQD1*^OE^, which had the highest GS content (Fig. 5). Furthermore, in vitro analysis with 10 μm BITC also demonstrated less double germ tubes in *ΔmfsG* compared with the wild type (Supplementary Fig. 3).

### *mfsG* confers tolerance to BITC in yeast

**Fig. 6:**
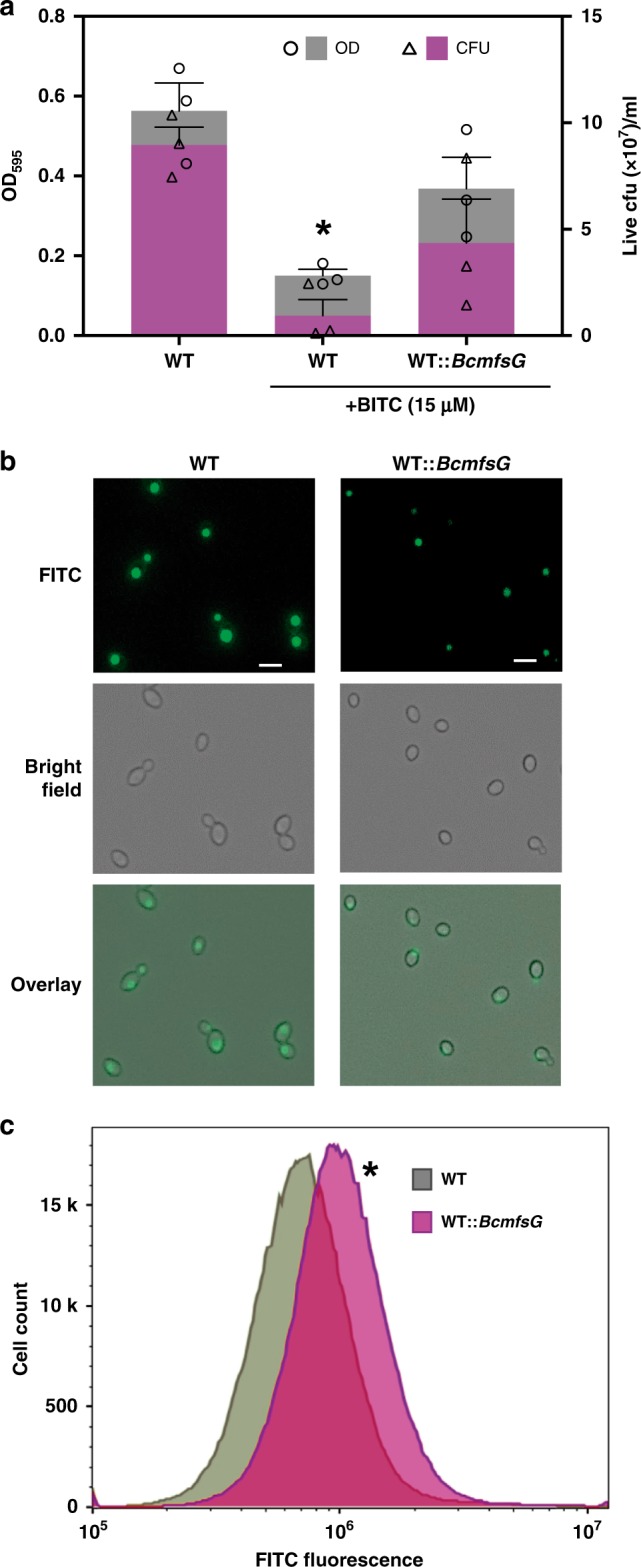
Ectopic expression of *B. cinerea mfsG* and efflux study in yeast. **a** *Saccharomyces cerevisiae* wild-type cells (WT), and cells expressing *BcmfsG* (WT::*BcmfsG*) were grown with or without 15 µm BITC in liquid media. OD and live colony-forming units (cfu) were measured after 48 h. Data are displayed as mean values (± SE). Asterisk denotes significant difference by Student’s *t* test (*P* *<* 0.001), *n* = 3. **b** *S. cerevisiae* WT and WT::*BcmfsG* cells were grown in liquid media with FITC and monitored under a fluorescence microscope 2 h later. **c** Fluorescence intensity was measured in WT as compared with WT::*BcmfsG* cells 2 h after exposure to FITC using flow cytometry. Asterisk denotes significant difference by Student’s *t* test (*P* *<* 0.001), *n* = 100,000 cells. Scale bars are 10 μm. Source data are provided as a Source Data file

To further study the functionality of *mfsG*, we determined its ability to induce tolerance to BITC in yeast cells. Wild-type yeast cells were highly susceptible to BITC; their growth was suppressed in the presence of 15 μm BITC, as determined by the optical density of the live culture and the number of colony-forming units (Fig. 6a). Interestingly, yeast cells expressing *B. cinerea mfsG* (WT::*BcmfsG)* were highly resistant to BITC (Fig. 6a). This was also evident in the transformed yeast cells’ ability to reach their exponential stage 10 h earlier than the wild-type cells under exposure to BITC (Supplementary Fig. 4). Furthermore, WT*::BcmfsG* yeast cells accumulated 40% less FITC than the wild-type yeast cells (Fig. 6b, c).

### Structural analysis of *mfsG*-encoded protein and BITC-binding analysis

**Fig. 7:**
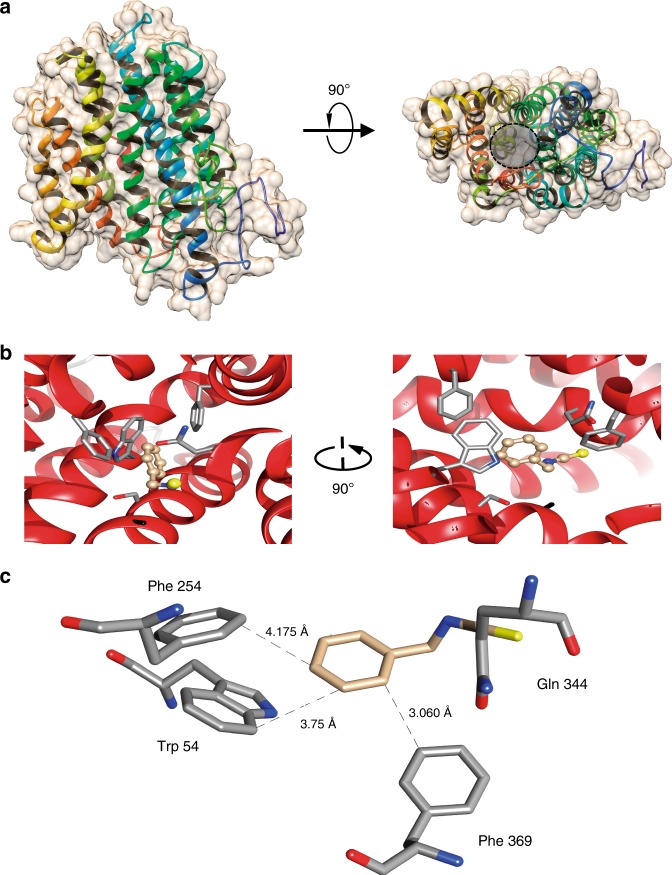
MFS motif tertiary structure and docking analysis of *B. cinerea mfsG*. **a** MFS tertiary structure viewed from the side of the membrane, and rotated through 90°; the conserved 12 transmembrane (TM) α-helix fold is arranged into two 6 TM helix bundles forming a cavity (marked with dashed circle) at the interface between them that is accessible to either the cytoplasm or extracellular region, depending on the conformational state. **b** Molecular docking between BITC molecule and MFS revealed a putative binding site formed by residues located in the internal channel of MFS. The proposed binding region is formed by the aromatic rings of residues Trp54, Phe254, Gln344, and Phe369. **c** Distances were measured between the BITC molecule and side chains of these residues, where the biggest distance was 4.175 Å with Phe254

**Fig. 8:**
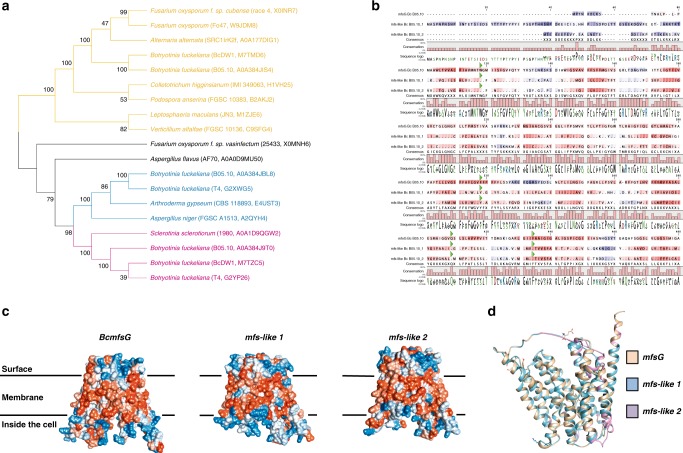
Phylogenetic and structural analysis of *mfsG* and *mfs-like* proteins. **a** Phylogenetic analysis was performed using amino-acid sequences of MFS proteins with at least 40% identity to *mfsG* from different fungi. All of these sequences were clustered into two clearly differentiated clades. One group contained B05.10 *mfsG* and similar proteins (yellow). The other clade consisted of three subclades: one of them (blue) contained B05.10 *mfs-like1* and its homologs, and another (pink) contained B05.10 *mfs-like2* and its homologs and a third that do not contain B05.10 homolog (black). Isolate name and protein ID are in parentheses. Bootstrapping (1000 replicates/iterations) was used to generate the phylogenetic tree with the neighbor-joining algorithm in MEGA7. **b** Alignment of amino-acid sequence of three MFSs from *B. cinerea* B05.10 (*mfsG*, *mfs-like1,* and *mfs-like2*). Residues located in the proposed binding site to ITCs are highlighted (green triangles). **c** Three-dimensional structure and hydrophobicity representation of *mfsG*, *mfs-like1*, and *mfs-like2* from *B. cinerea* B05.10. **d** Comparison of three-dimensional structures of *mfsG*, *mfs-like1*, and *mfs-like2*

*B. cinerea mfsG* protein encodes 431 amino acids with predicted molecular mass of 46 kDa and 12 putative transmembrane domains (Fig. 7a and Supplementary Fig. 5). Three-dimensional structure modeling of *mfsG* protein using the Phyre2 software obtained a 100% confidence alignment with a template for MFS general substrate transporters^32^. When *mfsG* structure was viewed from the side of the membrane, and rotated through 90°, it suggested that the conserved 12 transmembrane (TM) α-helix fold is arranged into two 6 TM helix bundles, forming a cavity at the interface between them that is accessible to either the cytoplasm or extracellular region, depending on the conformational state (Fig. 7a, Supplementary Fig. 5). When looking at the tertiary *mfsG* structure’s hydrophobic interactions, we observed a hydrophobic area inside the membrane, whereas the hydrophilic area was outside, either on the cell surface or inside the cell (Fig. 8c). We further performed molecular docking to map the interactions between the BITC molecule and *mfsG* to identify a putative binding site for this compound. This putative binding site was formed by residues located in the internal channel of *mfsG*. The proposed binding region was formed by the aromatic rings of residues Trp54, Phe254, Gln344, and Phe369 (Fig. 7b). Distances were measured between the BITC molecule and the side chains of these residues: the biggest distance was 4.175 Å with Phe254, and it was bound by one hydrogen bond to Trp54 (0.86 Å) (Fig. 7c). Molecular docking was also performed using PITC and PhITC (Supplementary Fig. 6a, b). PITC was bound by one hydrogen bond to Trp54 (2.63 Å), whereas PhITC was bound by one hydrogen bond to Arg290 (2.82 Å), both in the central channel of *mfsG*. Molecular docking using FITC showed that it is bound by one hydrogen bond to Trp54 (2.325 Å) in *mfsG* (Supplementary Fig. 6c). The thermodynamic parameters, Full fitness and free energy (ΔG), provided by SwissDock clearly indicated that the ITCs can bind tightly to *mfsG* (Table 1). Furthermore, we demonstrated molecular docking of other GS-breakdown products, such as nitrile molecules, to *mfsG*. Although 5-(methylsulfanyl)pentanenitrile bound to *mfsG* by two hydrogen bonds, one to Trp54 (2.08 Å) and the other to Arg290 (2.47 Å) (Supplementary Fig. 6d), benzonitrile was bound by one hydrogen bond to Trp54 (2.25 Å) (Supplementary Fig. 6e), but the high free energy needed to form the hydrogen bond suggested a strongly non-spontaneous binding process for both compounds (Table 1). Molecular docking with benzyl cyanide, another breakdown product of GS, revealed no binding sites to *mfsG* that also supported by the high free energy estimated (Table 1). A phylogenetic analysis using MFS proteins that share over 40% similarity to *mfsG* was performed (Fig. 8a; Supplementary Table 1). Sequence alignment revealed the presence of two well-represented clusters in the neighbor-joining phylogram. The first cluster contained *mfsG* and a closely related protein from *B. cinerea* BcDW1, a putative riboflavin transporter, as well as other closely related proteins. The analysis showed the existence of two other *mfs-like* proteins in *B. cinerea* B05.10 (gene IDs: BCIN_03g02910 and BCIN_02g05130), belonging to a separate cluster of *mfsG*, with homologs in *B. cinerea* T4, *B. cinerea* BcDW1 and their closest relative *Sclerotinia sclerotiorum*. Structural comparison of these three protein sequences from *B. cinerea* B05.10 showed that they have characteristics of MFS motifs and transmembrane domains in common (Fig. 8b, d), but they clearly differ in many residues along the amino-acid sequence, such as the aromatic residues that compose the core channel of *mfsG*; one of the missing residues was Phe254, which seems to be involved in GS-derivative binding to *mfsG*, as predicted by the molecular docking simulation. Nevertheless, thermodynamic analysis demonstrates lower possibility for binding (Supplementary Table 2) and no binding sites were found when molecular docking was carried out using BITC and *mfs-like* proteins from *B. cinerea* B05.10, T4 and *S. sclerotiorum*. This suggests that *B. cinerea* B05.10 *mfsG* is specific for GS-derivative detoxification ^33–36^.

**Table 1:** Thermodynamic parameters of molecular docking of ITCs to *mfsG*

	ΔG^0^ (Kcal mol^−1^)^a^	Full Fitness (Kcal mol^−1^)^b^
BITC	− 17.65	− 1392
PITC	− 11.8	− 1384
PhITC	− 6.42	− 1389
FITC	− 6.24	− 1378
5-(Methylsulfanyl)pentanenitrile	− 27.6	− 1402
Benzonitrile	0.68	− 1365
Benzyl cyanide	2.11	− 1367

^a^Free Gibbs energy ^b^ Score of docking analysis

## Discussion

Many studies have shown that the interaction between fungal pathogens and their host plant is mediated by secondary metabolites and corresponding pathogen tolerance. However, those studies focused mainly on the mechanisms of secondary metabolite detoxification and little data, if any, have been accumulated on the role of MFS transporters as key components in plant–pathogen interactions. In previous study, we showed that *A. thaliana* mutants with low GS content are more susceptible to *B. cinerea*^6,25^, other works also suggested that *B. cinerea* has variable sensitivity to GSs, as demonstrated by the enhanced susceptibility to *B. cinerea* of *cyp79B2/B3* compared with the wild type^6^. Thus, our and others’ studies show that *Arabidopsis*–*Botrytis* interactions are influenced at the metabolic level by variations in toxin production in the host and sensitivity in the pathogen. In the current study, we successfully showed that the MFS transporter *mfsG* is a crucial component contributing to *B. cinerea* virulence on GS-containing Brassicaceae plants such as *A. thaliana*. We demonstrated that *mfsG* expression was upregulated upon exposure to ITCs and on GS-containing plants (Fig. 1 and 4). We found a high correlation between linear mycelium inhibition and relative expression of *mfsG* in response to different concentrations of BITC (Pearson’s *r* = 0.9958). Resistance to antifungal compounds is commonly regulated at the transcriptional level, as shown by the upregulation of many transporters following exposure to specific toxins. Upregulation of transporters in plant pathogens indicates their role in the excretion of specific and non-specific host toxins as a protective strategy against plant defense compounds. For example, transcriptomic analysis of the Brassicaceae pathogen *Alternaria brassicicola* exposed to ITCs suggested that protection against Brassicaceae metabolites involves mechanisms aimed at limiting their intracellular accumulation, such as melanin biosynthesis and drug efflux.

Here we showed that *mfsG* is involved in ITC efflux, as suggested by the excess accumulation of FITC in Δ*mfsG* hyphae as compared with the wild type (Fig. 3). Plant pathogens use several modes of action to detoxify phytoalexins and phytoanticipins. General detoxification mechanisms such active efflux is powerful particularly in a broad host range pathogens such as *B. cinerea*. Several reports support our findings that *B. cinerea* uses transporters as a detoxification strategy for toxic compounds and plant secondary metabolites. For example, the *B. cinerea* ABC transporter *BcatrB*, involved in camalexin^14^ and resveratrol detoxification, confers pathogenicity on *Arabidopsis* and grapevine plants, respectively^10^. Moreover, it is also involved in detoxification of antibiotics and fungicides such as fludioxonil^37^ and phenazine^11^. Other transporters, such as *BcatrD* and *Bcmfs1*, have also been reported to be involved in azole fungicide transport.

We further demonstrated reduced virulence of *B. cinerea* mutant Δ*mfsG* on wild-type *A. thaliana* and enhanced susceptibility of the *cyp79B2/B3* mutant compared with *Col-0* when inoculated with Δ*mfsG* (Fig. 4). Fitness of Δ*mfsG* was also reduced in vitro and *in planta*, as verified by the lower frequency of double germ tubes (Fig. 5 and Supplementary Fig. 3). MFS transporters have been previously implicated in *Cercospora nicotianae* virulence, causing frog-eye disease on tobacco, where disruption of the *CTB4* gene displayed a drastic reduction in cercosporin secretion and a significant reduction in fungal virulence^38^. On the other hand, although the *Mycosphaerella graminicola Mfs1* confers resistance to azoles and cycloheximide, whereas its inactivation abolishes its resistance to fungicides^39^, the virulence on wheat seedlings of Δ*MgMfs1* was not affected^40^.

The slightly reduced virulence of Δ*mfsG* mutants on tomato plants (Supplementary Fig. 7) suggests that *mfsG* might also be involved in the detoxification of other toxic compounds, similar to *BcatrB*, *BcatrD*, and *Bcmfs1*. The simultaneous resistance to a number of unrelated compounds is called multidrug resistance (MDR). It can play a major role in the resistance of plant pathogens, including *B. cinerea*, to agricultural fungicides. Therefore, identification and characterization of drug transporters in plant pathogens is crucial to producing effective management strategies for fungicide resistance.

MDR families of transporters are widely distributed in different types of cells. However, only two are found in all classes of organisms: the ABC and MFS^41–44^. In general, MFS transporters are ubiquitous transporters that can only transport small solutes in response to chemiosmotic ion gradients. However, in some cases, MFS transporters are also believed to act as drug transporters using a proton gradient, thus conferring multidrug and multixenobiotic resistance in bacteria, fungi, and more-complex eukaryotes^41,42^. Nevertheless, in general, MFS transporters are more substrate specific compared with ABC transporters^45,46^. For example, the phenotype of *B. cinerea* MDR2, a field strain that is resistant to several fungicides, is correlated with increased drug-efflux activity via overexpression of the *mfsM2* transporter^47,48^. However, using the MDR2 isolate, we could not see any resistance to BITC (Supplementary Fig. 8), supporting our hypothesis of *mfsG*’s specificity for GS-breakdown products. Furthermore, the drug-efflux MFS transporters can also confer MDR in mammalian pathogens; for example, *Mdr1* from *Candida albicans* has been reported as a MDR transporter that confers resistance to fungicides such as fluconazole and ketoconazole^41,49,50^. Expression of *CaMdr1* confers MDR also to the model yeast *S. cerevisiae*^51^. We also demonstrated that *B. cinerea mfsG* can confer tolerance to BITC in *S. cerevisiae* yeast cells by providing ITC efflux ability (Fig. 6).

Plant pathogens must protect themselves, especially during infection when they are likely to encounter host defense mechanisms. Therefore, membrane transporters are key elements in toxic compound detoxification. They play a role in counteracting the physiological impact of antimicrobial host defense compounds, as evidenced by the large number of MFS transporters in the genomes of necrotrophic fungal plant pathogens such as *Sclerotinia sclerotiorum* and *B. cinerea*: 218, 286, and 282 genes in *S. sclerotiorum*, *B. cinerea* T4 and *B. cinerea* B05.10, respectively. Genome comparison showed that many *B. cinerea* genes, absent in *S. sclerotiorum*, had no orthologues in other fungi, indicating that gene expansion in *B. cinerea* leads to the observed differences between plant–pathogen genomes^52^. There are also variations among the different *B. cinerea* isolates in their response and resistance to GSs and in their transporters’ availability^25^ (Fig. 8). BLAST analysis with *mfsG* transporter revealed two *mfs-like* genes in B05.10 that share over 40% identity with *mfsG*. These two *mfs-like* genes were also found in other *B. cinerea* isolates (T4 and DW1) and in *B. cinerea*’s close relative *Sclerotinia sclerotiorum* (Fig. 8). Although isolate DW1 had a homolog of *mfsG*, T4 only had the *mfs-like* homologs (Fig. 8). Docking analysis demonstrated that only *mfsG* can efficiently bind GS-breakdown products (Table 1), whereas *mfs-like* proteins have lower probability to bind ITCs (Supplementary Table 2). This may also support the hypothesis of *mfsG*’s specific involvement in GS detoxification (Table 1 and Fig. 7). *B. cinerea* T4 and *S. sclerotiorum* may have evolved other detoxification pathways, such as using glutathione S-transferase proteins or other transporters like *Alternaria brassicicola*^53–55^. It is clear that the natural function of *mfsG* is to protect *B. cinerea* against aromatic fungitoxic compounds during the pathogenic process. Other studies have postulated that ABC and MFS transporters can also function as effectors or pathogenicity factor secretion, thereby affecting fungal virulence^56–59^. Furthermore, Dos Santos and colleagues' studies on MFS transporters in yeast suggest that these transporters can also provide drug resistance by indirect regulation of the stress response and control of membrane potential. Plant–pathogen transporters may have similar effects.

Finally, we provide evidence of a *B. cinerea* MFS transporter’s involvement in GS detoxification and its function as a virulence factor. Further analysis is needed to reveal whether the *B. cinerea mfsG* transporter involved in detoxification of other compounds, and whether it also acts indirectly to provide tolerance to the stress response during plant infection.

## Methods

### Plant material and growth conditions

*A. thaliana* plants: wild-type Columbia (*Col-0*), *IQD1*^*OE*^, *tgg1/2,* and *cyp79B2/B3*, were grown at 22 °C and 60% relative humidity under fluorescent and incandescent light at a photofluency rate of ~ 120 μmol m^−2^ s^−1^ and a 12/12 h photoperiod. *Arabidopsis* seeds were stratified on moist soil mix at 4 °C for 2 days and then transferred to a growth chamber for 4 weeks.

### Fungal strains—growth and inoculation of plant material

*B. cinerea* isolates B05.10 were cultured on potato dextrose agar (PDA, Difco) in a controlled-environment chamber at 22 ^o^C in the dark or under illumination with fluorescent and incandescent lights at a photofluency rate of 120 μmol m^−2^ s^−1^ and 12/12 h photoperiod. Conidia were harvested from the light-grown culture in sterile distilled water containing 0.001% (v per v) Triton X-100 (J.T. Baker) and filtered through a 40-µm cell strainer to remove remaining hyphae. For inoculation, the conidial suspension was adjusted to 10^5^ conidia ml^−1^ in half-strength filtered (0.45 μm) grape juice (100% pure organic). Detached leaves from 4-week-old plants were placed on water–agar (1% w per v) trays and each leaf was inoculated with 4-μl droplets of conidial suspension. Trays were covered with a plastic dome and placed in the growth chamber. At 48 h post inoculation, leaves were photographed and lesion sizes were analyzed using ASSESS 2.0 image analysis software for plant disease quantification (APS Press).

### Yeast strain and growth conditions

*S. cerevisiae* BY4742 (*MAT*α, *his*3Δ1, *leu*2Δ0, *lys*2Δ0, *ura*3Δ0) (kindly provided by Dr. Juergen Stolz, Technical University of Munich, Germany) yeast cells were grown in YPD (2% peptone, 2% glucose/galactose, 1% yeast extract, w per v) or YNB (2% galactose/glucose and 0.67% yeast nitrogen base, w per v) medium at 30 °C.

### ITC inhibition of radial mycelial growth in vitro

The inhibitory effect of ITCs on mycelial growth of *B. cinerea* (B05.10), *ΔmfsG* and the complemented mutants was tested by placing a 6-mm diameter plug from 3-day-old *B. cinerea* colony margins grown in the dark at 22 ^o^C on a freshly prepared PDA plate containing different concentrations of ITCs. The diameter of radial mycelial growth (D, in mm) was recorded at 24 h for treatments (Dt), and controls (Dc). The fungitoxic effects of ITCs were calculated as percentage of growth inhibition (GI) as: GI(%) = (*D*c—*D*t)*/D*c × 100. PITC, BITC and PhITC were obtained from Sigma-Aldrich. Stock solutions of each ITC were prepared in methanol.

### RNA extraction, cDNA synthesis, and qRT-PCR

RNA was extracted from *B. cinerea* mycelium after 48 h growth on PDA supplemented with different ITCs using Plant/Fungi Total RNA Purification Kit (Norgene Biotek Corp). For fungal RNA in planta, total RNA was extracted from 100 mg leaves (12 leaves from three different plants for each treatment) 60 h post inoculation with *B. cinerea* using Tri-Reagent (Sigma-Aldrich) according to the manufacturer’s instructions.

RNA was treated with DNase using the TURBO DNA-Free Kit (Ambion) according to manufacturer’s directions. DNA-free RNA (0.5–1 µg) was reverse-transcribed to cDNA using the High-Capacity cDNA Reverse Transcription Kit (Applied Biosystems) according to the manufacturer’s instructions. The cDNA was used as a template in qRT-PCR with Fast SYBR Green Master Mix (Applied Biosystems) and a StepOne real-time PCR machine (Applied Biosystems) according to the manufacturer’s protocol. The thermal cycling program was as follows: 94 °C for 2 min; 40 cycles of 94 °C for 15 s, 67 °C for 45 s, and 72 ^o^C for 1 min; final extension step at 72 ^o^C for 10 min. Relative fold change of mRNA levels of *mfsG* (using primers MFS-F and MFS-R), were normalized to *B. cinerea* actin (BC1G_08198; using primers Actin-F and Actin-R) and calculated by the 2^−ΔΔCt^ method using StepOne Plus analysis software version 2.3 (Applied Biosystems). The primer sequences are shown in Supplementary Table 3.

### Construction of *mfsG*-knockout cassette

The *mfsG* (BC1G_15286; BCIN_06g00026)-knockout cassette was constructed based on Multiple Gateway Three Fragment Vector Construction technology (Invitrogen)^60,61^. The Hyg cassette (2935 bp) was amplified from the pOliHP plasmid using primers attB1hygF and attB2hygR (Supplementary Table S3) ^62,63^. This fragment was cloned into entry clone plasmid pDONR221 by a BP recombination reaction. PCR fragments of 504 bp were amplified from the 5´ and 3´ regions of *mfsG*, respectively, using primers attB3’F and attB3’R for the 5´ region and primers attB5’F and attB5’R for the 3´ region (Supplementary Table 3). These fragments were individually cloned into entry clone plasmid pDONR P4-P1R and pDONR P2R-P 3, respectively, by BP recombination reaction. After sequencing all three plasmids, a MultiSite Gateway LR recombination reaction was performed between multiple entry clones and the pDEST™R4-R3 vector to generate an expression clone. This final plasmid was named pDEST-*ΔmfsG*, and was linearized by enzymatic digestion using *Dra*I. The replacement cassette was purified by ethanol precipitation and resuspended in sterile water supplemented with 0.01% (v per v) Silwet L-77 surfactant (Agri-Turf Supplies) prior to transformation.

### Plasmid construction for complementation in *B. cinerea*

The primers MFSpOliNat1-F and MFSpOliNat1-R (Supplementary Table 3) were used to amplify the full-length wild-type *mfs* gene from genomic DNA of *B. cinerea* B05.10. This fragment was cloned into the plasmid pOliHP, replacing the *hph* gene, by restriction-free (RF) cloning method, then *Dpn*I-treated and finally transformed into *E. coli* DH5α, resulting in the plasmid pOliMFS. The *nat1* gene, amplified from the plasmid pNAN-OGG^64^ using the primers Nat1pOli-F and Nat1pOli-R (Supplementary Table 3), was cloned into the plasmid pOliMFS, generating pOliMFSNat1 which was used for transformation of *B. cinerea* Δ*mfsG* to generate *mfsG*-complementation^65^.

### Transformation of *B. cinerea*

*B. cinerea* (B05.10) sclerotial transformation was performed. *B. cinerea* sclerotia were collected from mature colonies grown on PDA plates for 10 days at 18 °C. Sclerotia (50–60) were disinfected by three washes with 1% sodium hypochlorite, followed by three washes with sterilized purified water, and were completely dried prior to transformation on sterile Whatman filter paper in a biological hood. The dried sclerotia were wounded by making a hole in the middle of the sclerotium (without penetrating through) with a sterile needle (21 G), followed by application of 5 μl DNA solution of linearized pDEST-Δ*mfsG* (a total of 0.5 μg) supplemented with 0.01% Silwet L-77. Vacuum (~ 600 mbar) was applied for 10 min, the solution was fully absorbed, and sclerotia and were further dried in a biological hood. Sclerotia were placed on PDA plates (10 ml plate^−1^) and incubated at 24 °C for 48 h of recovery. The growing hyphae were then transferred onto new plates containing PDA supplemented with 25 μg ml^−1^ Hygromycin B (TOKU-E). Putative mutants were purified by hyphal tip transfer to new selection plates with increasing hygromycin concentrations of 5 μg ml^−1^ to 40 μg ml^−1^ (each concentration was applied five times). The mutants were grown on PDA supplemented with hygromycin and genomic DNA purification was performed using the MasterPure^TM^ Yeast DNA Purification Kit (Epicentre) according to the manufacturer’s protocol. To verify transformation, we performed PCR analyses on DNA extracted from putative transformants using hygromycin cassette primers Hyg-F and Hyg-R (Supplementary Table 3 and Supplementary Fig. 9).

All PCR analyses were performed in 0.25-ml tubes containing PCR reagent (ReddyMix^®^, Thermo Fisher Scientific Inc.) with 5 pmol of primers, 12.5–25 ng template DNA and sterile purified water to a final volume of 25 μl. PCR was carried out in an MJ Mini Thermal Cycler (BioRad). Enzyme activation was carried out at 95 °C for 4 min, followed by denaturation for 30 s at 95 °C, and annealing at 55 °C for 40 s, elongation at 72 °C for 40 s for 40 cycles, and 10 min of elongation at 72 °C.

For knockout mutant complementation, *B. cinerea* Δ*mfsG* mutants were transformed by conidial electroporation. Fresh spore suspension was prepared as described above (Fungal strains—growth and inoculation of plant material), centrifuged and resuspended in 50 ml of potato dextrose broth (PDB, BD Biosciences). The spore suspension was then incubated for 4 h at 25 °C and 100 rpm. Germinating conidia were collected by centrifugation at 5000 rpm for 10 min and resuspended in 20 ml of ice-cold KC buffer (0.6 m KCl, 50 mm CaCl_2_); this step was repeated twice to wash the cells. Aliquots of 120 µl (1.2 × 10^6^ spores) were placed in pre-chilled 0.2-cm electroporation cuvettes and kept on ice. The conidial suspension was gently mixed with 30 µg of linearized plasmid and incubated on ice for 10 min. Transformation was conducted by application of electroporation pulses two pulses at 1.70 kV, 800 Ω, 25 µF with an interval of 5 s using an electroporator (Eppendorf 2510 system)^66^. Conidia were then directly resuspended in 1 ml ice-cold KC buffer and placed on ice for 10 min. Conidial suspension was then mixed with 10 ml tempered SH agar (1 mm NaNO_3_, 0.6 mm sucrose, 5 mm Tris pH 6.5), poured on plates and incubated at 25 °C. After 48 h, the plates were covered with a 10 ml layer of SH agar amended with 50 µm BITC and/or 40 µm nourseothricin (Nat) and the two-layer plates were incubated at 25 °C for 2 weeks until the transformants grew through the agar. The putative transformants were transferred to PDA plates where the concentration of selection agents was increased progressively to 100 µm for BITC and 80 µm Nat.

### Molecular characterization of Δ*mfsG* mutants and complemented mutants

Putative *B. cinerea* Δ*mfsG* mutants were grown on PDA plates amended with 40 µg ml^−1^ hygromycin. Genomic DNA was extracted using plant/fungi Total DNA Purification Kit (Norgene Biotek Corp.) and RNA was extracted from small amounts of mycelium as described in the RNA extraction section. To confirm the knockout mutation, the expression of *mfsG* was analyzed by RT-PCR amplifying a 300-bp fragment with primer pairs MFS-F and MFS-R (Supplementary Fig. 9 and Supplementary Table 3). In addition, the presence of *hph* gene promoter, POliC, was confirmed in putative mutants amplifying a 552-bp fragment by PCR from genomic DNA with the primers POliC-F and POliC-R (Supplementary Fig. 9 and Supplementary Table 3). *B. cinerea* β-tubulin (*Bctub2*) was used as a control for cDNA and DNA by amplifying a 500-bp fragment with the primers tub2-F and tub2-R (Supplementary Fig. 9 and Supplementary Table 3). PCR amplifications were conducted using with proofreading Phusion High-Fidelity DNA Polymerase (Thermo Fisher Scientific). The amplification conditions consisted of an initial denaturing step at 98 °C for 2 min, followed by 35 cycles of 94 °C for 1 min, 52 °C for 1 min, 72 °C for 1 min, and a final elongation step at 72 °C for 5 min.

Putative complemented mutants were maintained in PDA plates amended with 40 µm Nat. To confirm the complementation, the presence of *mfsG* cassette was screened by PCR as described above. In addition, the presence of Nat resistance gene (*Nat1*) was confirmed using the primers nat-F/nat-R (Supplementary Fig. 9 and Supplementary Table 3). PCR conditions were as described above.

### GI of Δ*mfsG* mutants and complemented mutants on BITC

The wild-type strain *B. cinerea* B05.10, Δ*mfsG* mutants, and complemented mutants were cultured on PDA amended with a range of different concentrations of BITC: 0, 75, 100, 150, 200 μm. Mycelial growth was recorded after 72 h and GI was calculated relative to fungi cultured on PDA alone.

### Germination and double germ tube test in planta

Different genotypes of *A. thaliana* were inoculated with a spore suspension (10^6^ spore ml^−1^) of *B. cinerea* wild-type, Δ*mfsG* mutants, and complemented mutants. Detached leaves were dipped in spore suspension for 1 min, and placed on water–agar trays (1%); 24 h post inoculation, the leaves were cleared in boiling absolute ethanol for 20 min in a water bath, followed by a final wash in glycerol:lactic acid:water (1:1:1 v per v) overnight. The leaves were then incubated for 2 min in aniline blue (0.2% w per v) followed by rinsing in distilled water. Finally, germination rate and number of double germ tubes were calculated under a bright-field microscope.

### Efflux study in *B. cinerea* using FITC

*B. cinerea* isolates were grown on PDA containing 10 µm FITC (Sigma-Aldrich) for 4 days at 22 °C in the dark. Samples of *B. cinerea* hyphae were analyzed by confocal microscopy (Zeiss LSM-510). Fluorescence inside the cells and in the media was quantified on confocal photomicrographs using Image J software (NIH).

### Pathogenicity test of Δ*mfsG* and complemented mutants

The wild-type *B. cinerea* strain B05.10, Δ*mfsG* and full-length complemented mutants were screened by plant infection assay^25^. Detached leaves of different transgenic lines of *A. thaliana* or mutants impaired in GS production were placed on trays of water–agar media (1%) and inoculated with 4-μl droplets of conidial suspension (10^5^ spore ml^−1^); 72 h post inoculation, lesions, were measured using the image analysis software ASSESS 2.0 for plant disease quantification. Leaves for pathogenicity study were taken from at least 10 different plants with random location in the growth room. All presented data are representative of at least three independent experiments with similar results.

### Heterologous expression of *B. cinerea mfsG* in yeast

The *mfsG* (BC1G_15286; BCIN_06g00026) open reading frame was amplified from *B. cinerea* genomic DNA with primers that added restriction sites adjacent to the start and stop codons (BcmfsG-F–*EcoR*I and BcmfsG-R–*Xho*I, respectively; Supplementary Table 3). The PCR products were ligated into pGEMT-easy (Promega), sequence-verified, and finally cloned downstream of the galactose-inducible *GAL1* promotor of the multicopy vector pYES2 (Invitrogen). pYES2–*BcmfsG* was used to transform *S. cerevisiae* yeast strain BY4742 by the lithium acetate procedure^67^. Yeast cells were grown in YPD (glucose) medium till reaches to mid-log phase. Cells were than harvested by centrifugation and washed with TE (10 mm Tris-HCl (pH 8.0) and 1.0 mm EDTA) and finally suspended in TE to the final concentration of 2 × 10^8^ cells ml^−1^. Suspended cells were mixed with an equal volume (0.5 ml) of 0.2 m metal ions (LiAc) to final volume of 1 ml and incubated for 1 h at 30 °C with shaking (140 rpm). In total, 100 µl of this mixed suspension incubated with 15 µl of a plasmid DNA solution (670 µg ml^−1^) at 30 °C for 30 min. An equal volume of 70% PEG 4000 dissolved in water was mix thoroughly with the cells and incubated at 30 °C for 1 h, followed by 5 min incubation at 42 °C. Cells were immediately cooled to room temperature. Washed twice with water and suspended to 1 ml of water. Selection of transformants was carried out on YNB minimal medium lacking uracyl (auxotrophic selection) and amended with thiamine to prevent possible toxic effects of plasmid expression.

### Yeast GI by BITC

The growth-inhibitory effect of BITC was tested using different strains of yeast: BY4742 (WT) and BY4742::*BcmfsG (WT::BcmfsG)*. Strains were grown in liquid and solid media. The yeast strains were grown in YPD medium containing galactose as a carbon source (Sigma-Aldrich) for 5 h (optical density at 595 nm [OD_595_] = 0.5). The yeast strains were diluted and 15 μm BITC was added to the minimal synthetic media for yeast (YNB + galactose). The yeast cells were grown at 30 ^°^C in a plate reader (TECAN, Infinite F200) and the OD_595_ was recorded (four reads per well in triplicate per sample) and registered for every well (48-well plate, 300 μl per well) every hour after shaking. In addition, samples were taken at different time points; the yeast were diluted and cultured on YPD (glucose) agar plates at 30 ^°^C. The colonies were counted and correlated to the results of the OD readings. The yeast cells were grown at 30 ^°^C for 2 days before colony counting and the results were analyzed.

### FITC efflux in yeast

Photomicrographic and quantitative FITC-efflux assays were performed as described by Preston et al.^68^ with the following adaptations. The yeast strains were grown in YPD medium containing galactose (Sigma-Aldrich) as a carbon source under constant agitation (150 rpm) for 5–8 h to an OD_595_ of 0.5. FITC (10 μg ml^−1^) was added along with 50 mm sodium citrate and cultures were incubated at 37 ^o^C for 30 min in the dark. The yeast cells where then washed three times with minimal synthetic medium YNB (galactose) and incubated at 30 ^o^C. Samples were taken after 2 h for fluorescence flow cytometry analysis (CytoFlex, Beckman) and for microscopy (EVOS FL AUTO, Thermo Fisher). For flow cytometry analysis no sorting or gating were done, 100% of the cells showed normal SSC-A/FSC-A distribution (Supplementary Fig. 10) and were included in the analysis using FlowJo software.

### Protein modeling and molecular docking

The “Protter server [http://wlab.ethz.ch/protter/start/]” was used to depict the *B. cinerea mfsG*-encoded protein’s secondary structure. “Phyre2 workspace [www.sbg.bio.ic.ac.uk/phyre2]” was used for automated protein tertiary structure homology modeling of this protein^32^. To identify potential binding sites of BITC (PubChem ID: 2346), PITC (PubChem ID: 69403), PhITC (PubChem ID: 7673), FITC (PubChem ID: 113298), 5-(Methylsulfanyl)pentanenitrile (PubChem ID: 93320), Benzonitrile (PubChem ID: 7505), and Benzyl cyanide (PubChem ID: 8794) to the *mfsG*-encoded protein, automated molecular docking and thermodynamic analysis were performed using the web-based “SwissDock program [www.swissdock.ch/docking]”^69^. SwissDock predicts the possible molecular interactions between a target protein and small molecule is based on the docking algorithm EADock DSS^70^. The docking was performed using the “Accurate” parameter at otherwise default parameters, with no region of interest defined (blind docking). Binding energies are estimated by using CHARMM (Chemistry at HARvard Macromolecular Mechanics), a molecular simulation program implemented within SwissDock software, and the most favorable energies are evaluated by FACTS (Fast Analytical Continuum Treatment of Solvation). Finally, the results on energies are scored and ranked by full fitness (kcal mol^−1^) and the spontaneous binding is exhibited by the estimated Gibbs free energy ΔG (kcal mol^−1^). The negative values of ΔG support the assertion that the binding process is highly spontaneous. Modeling and docking results were visualized using UCSF Chimera v1.8 software.

### Statistical analysis

When data were normally distributed and sample variances were equal, *t* tests were performed. In all other cases, Mann–Whitney Rank Sum test was performed. For multiple comparisons, one-way analysis of variance (ANOVA) was performed when the equal variance test was passed. In all other cases, one-way ANOVA on ranks was performed (Kruskal–Wallis or Tukey–Kramer honest significant difference test). Significance was accepted at *P* < 0.05.

### Reporting summary

Further information on research design is available in the Nature Research Reporting Summary linked to this article.

## Supplementary information

Supplementary Information

Reporting Summary

Source Data

## Data Availability

Data underlying Figs. 1, 2, 3b, 4, 5, 6a, 6c and Supplementary Figs. 2, 3b, 4, 7, 8, and 9 are provided as Source Data files. All other data are available from the corresponding author upon reasonable requests. *BcmfsG* accession number at NCBI is BCIN_06g00026 (old locus tag is BC1G_15286).
