# Supplementary material for: MFS transporter from Botrytis cinerea provides tolerance to glucosinolate-breakdown products and is required for pathogenicity
Source: Nat Commun. 2019 Jun 28;10:2886. doi: 10.1038/s41467-019-10860-3 (PMC6599007; doi:10.1038/s41467-019-10860-3)
Supplement: Supplementary file 1 — Supplementary Information [file 41467_2019_10860_MOESM1_ESM.pdf]

MFS transporter from *Botrytis cinerea* provides tolerance to glucosinolate-breakdown products and is required for pathogenicity

Vela-Corcia et al.,

SUPPLEMENTARY

Supplementary Methods

Phylogenetic analysis

The *mfsG* amino acid sequence was subjected to BLAST search using the UniProt knowledgebase (The UniProt Consortium, 2018). A number of different fungal MFS transporter sequences (**Supplementary Table 1**) were aligned using Clustal Omega[1] to create a phylogram representing the most parsimonious phylogenetic relatedness of *Botrytis cinerea mfsG* with other transporter sequences within the fungi. Bootstrap analysis (1000 replicates/iterations) was used to generate the phylogenetic tree with the neighbor-joining algorithm in MEGA 7 [2]. The transporter hits related to *mfsG* were initially identified using the B-Link web tool on the NCBI network.

RNA-Seq analysis

Transcriptional analysis was performed on *B. cinerea* (B05.10) hyphae growing on potato dextrose agar (PDA, AppliChem) amended with 75 μM benzyl isothiocyanate (BITC) as compared to PDA using RNA-Seq as described previously[3]. Total RNA was extracted (Plant/Fungi Total RNA Purification Kit; Norgen Biotek Corp.) from each sample 72 h postinoculation, and the quality and quantity were measured by running the sample on an Agilent Bioanalyzer 2100. The RNA-Seq libraries were prepared and validated and the cDNA samples were sequenced using HiSeq 2000 (Illumina). A total of 3 μg RNA per sample was used as the input material for the RNA sample preparations. The RNA-Seq libraries were prepared with the High-Capacity cDNA Reverse Transcription Kit (Applied Biosystems). Library validation was accomplished using Agilent DNA 1000 (Agilent Technologies) and Illumina Library Quantification (Kapa Biosystems) kits. The cDNA samples were sequenced using HiSeq 2000 at HT-Seq Technion (Israel). Differentially expressed gene sequences were annotated using Blast2GO software[4].

Germination and double germ tube test *in vitro*

PDA plates supplemented with 10μM BITC inoculated with a spore suspension (10^4 spore ml^{-1}) of *B. cinerea* wild-type, Δ*mfsG* mutant and complemented mutant and incubated at 25°c for 12 h. The spores were then incubated for 2 min in aniline blue (0.2% w/v) followed by rinsing in distilled water. Finally, number of double germ tubes were calculated under a bright-field microscope.

Supplementary Results

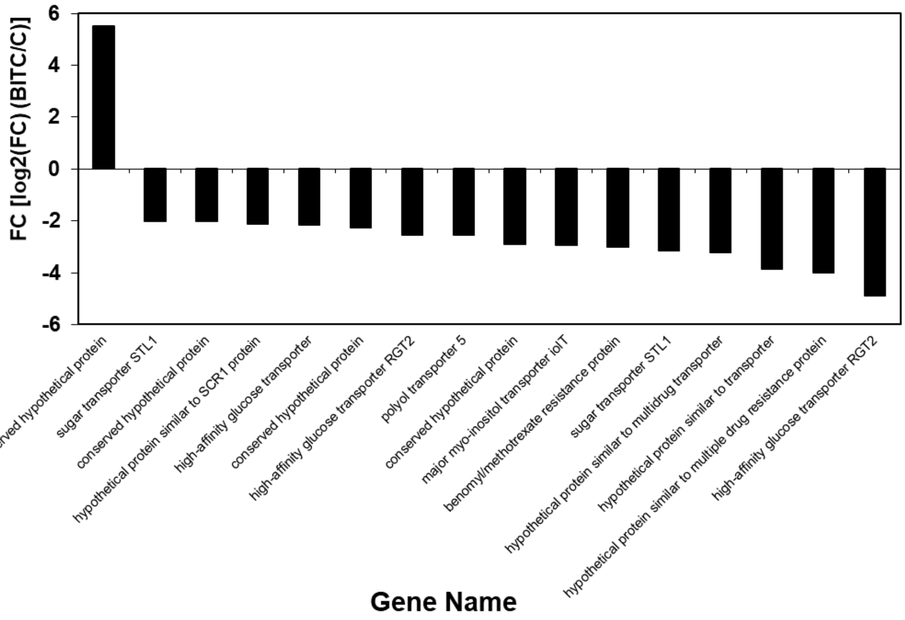

Supplementary Figure 1. Expression analysis of major facilitator superfamily (MFS) domain-enriched genes after BITC treatment. MFS domain enrichment analysis (pFam) was performed on genes that were differentially regulated after BITC treatment. The y-axis indicates the log2 fold change of genes with the MFS motif after exposure to 75 μM BITC as compared to untreated sample and the x-axis indicates the gene name.

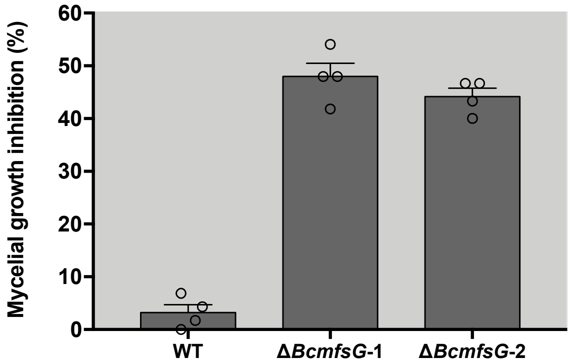

Supplementary Figure 2. Growth inhibition of Δ*mfsG* by BITC. The diameter of *B. cinerea* wild type (WT) and two independent deletion mutant (Δ*mfsG*) colonies was measured 48 h postinoculation on PDA plates supplemented with 10 μM BITC. Growth inhibition was calculated as percentage of colony diameter of *B. cinerea* grown on PDA with no BITC. Data are displayed as mean values (±SE), n>3. Source data are provided as a Source Data file.

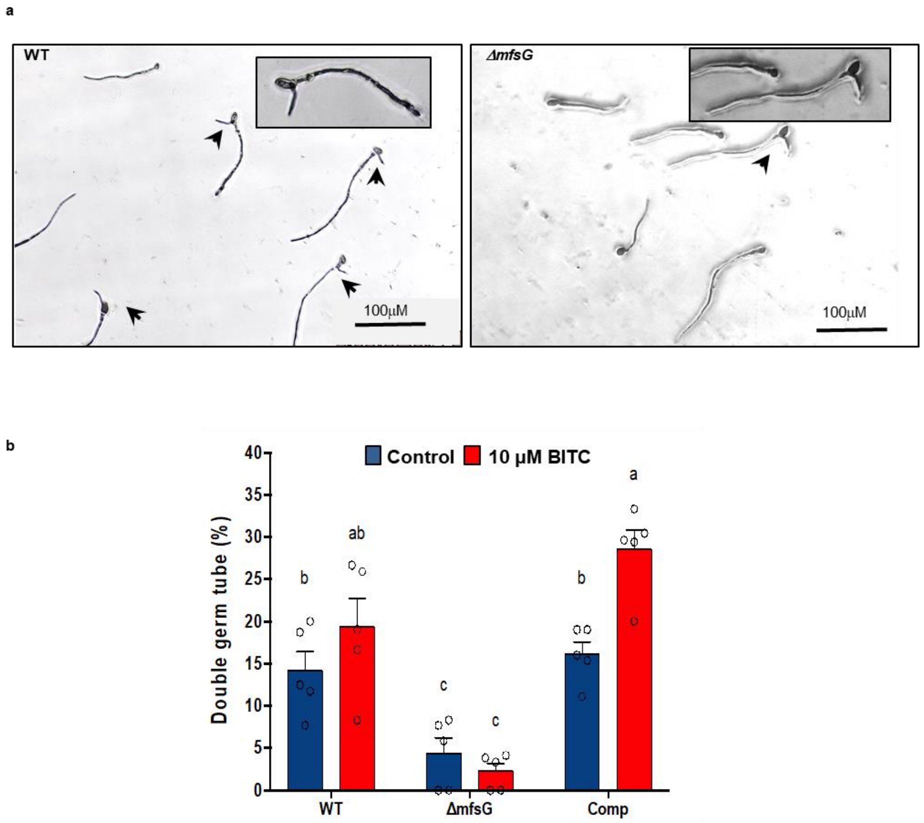

Supplementary Figure 3. Germination test of *B. cinerea mfsG*-knockout (Δ*mfsG*) and complemented mutants. **a** Double (marked with arrow head) and single germ tunes under light microscopy. **b** The number of germ tubes in germinating conidia was evaluated by bright-field microscopy 24 h after inoculation of PDA plates supplemented with 10 μM BITC as compared to control (PDA) with *B. cinerea* wild type (WT), knockout mutant (Δ*mfsG*) and complemented mutant (Comp). Data are displayed as mean

values (±SE). Different letters above the columns indicate significant difference at $P <$ 0.05, as determined by Tukey-Kramer HSD, n>98. Source data are provided as a Source Data file.

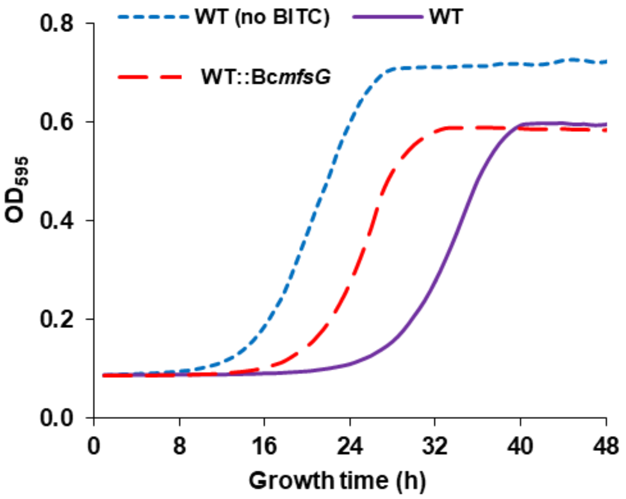

Supplementary Figure 4. Growth curves of yeast cells. *Saccharomyces cerevisiae* wild-type cells (*WT*) or *BcmfsG* expressing cells *(WT::BcmfsG*). All cells were grown in liquid media without (no BITC) or with 15 μM BITC. OD was recorded every hour for 48 h. Source data are provided as a Source Data supplementary file.

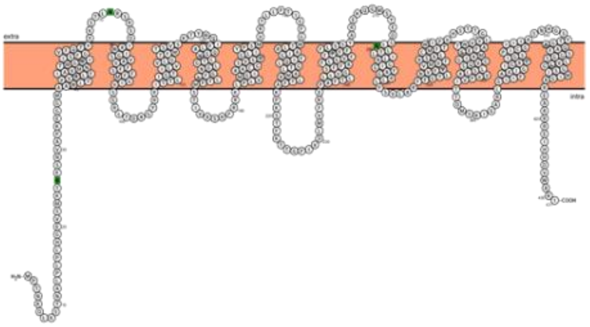

Supplementary Figure 5. *mfsG* protein structure. **a** MFS topology demonstrates 12 conserved transmembrane α-helixes.

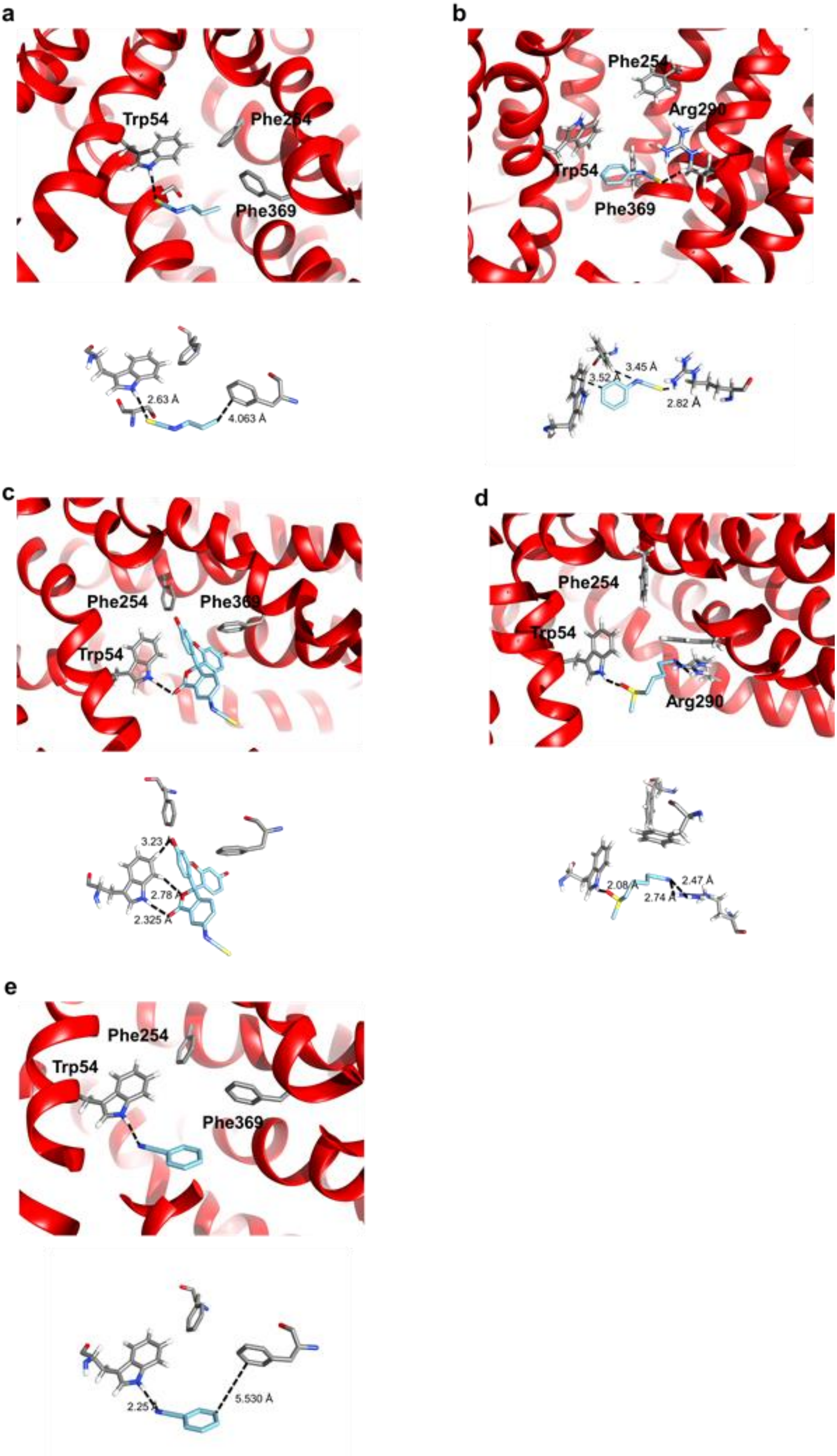
a
Trp54
Phe254
Phe369
2.63 Å
4.063 Å
b
Phe254
Arg290
Trp54
Phe369
3.45 Å
3.52 Å
2.82 Å
c
Phe254
Phe369
Trp54
3.23
2.78
2.325 Å
d
Phe254
Trp54
Arg290
2.08 Å
2.47 Å
2.74 Å
e
Phe254
Trp54
Phe369
2.25
5.530 Å

Supplementary Figure 6. Molecular docking of ITCs and nitriles to *B. cinerea* B05.10 *mfsG*. **a** Propyl isothiocyanate (PITC) (PubChem ID: 69403) is proposed to bind *mfsG* via one hydrogen bond (dashed line) that involves residue Trp54 (2.636 Å). **b** Phenyl isothiocyanate (PhITC) (PubChem ID: 7673) is proposed to bind *mfsG* via one hydrogen bond (dashed line) that involves residue Arg290 (2.818 Å). **c** Fluorescein isothiocyanate (FITC) (PubChem ID: 113298) is proposed to bind *mfsG* via one hydrogen bond (dashed line) that involves residue Trp54 (2.325 Å). **d** 5-(Methylsulfanyl)pentanenitrile (PubChem ID: 93320) is proposed to bind *mfsG* via two hydrogen bonds that involve residues Trp54 (2.083 Å) and Arg290 (2.478 Å). **e** Benzonitrile (PubChem ID: 7505) is proposed to bind *mfsG* via one hydrogen bond (dashed line) that involves residue Trp54 (2.246 Å). The insets for each panel represent the ligand and distances to the closest residues of *mfsG*.

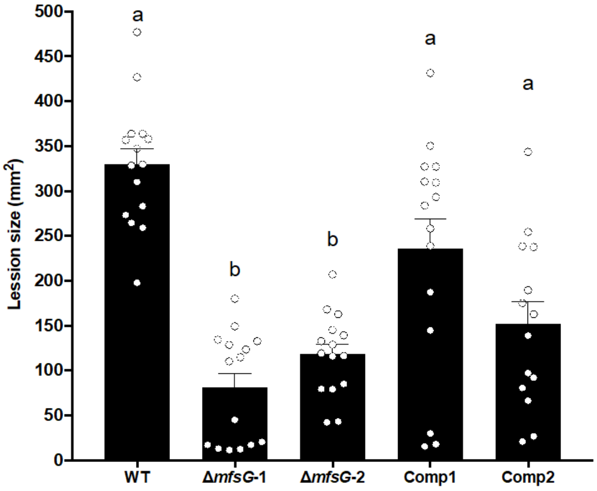

Supplementary Figure 7. Pathogenicity test of *B. cinerea* knockout mutants and complemented mutants on tomato leaflets. Lesion size was measured 72 h after inoculation with *B. cinerea* (WT, ΔmfsG and comp.). Average lesion size of 15 leaves of each isolate is presented together with the standard errors for each average. Different letters or asterisks above the columns indicate significant difference at $P < 0.05$, as determined by Kruskal–Wallis test. Source data are provided as a Source Data file.

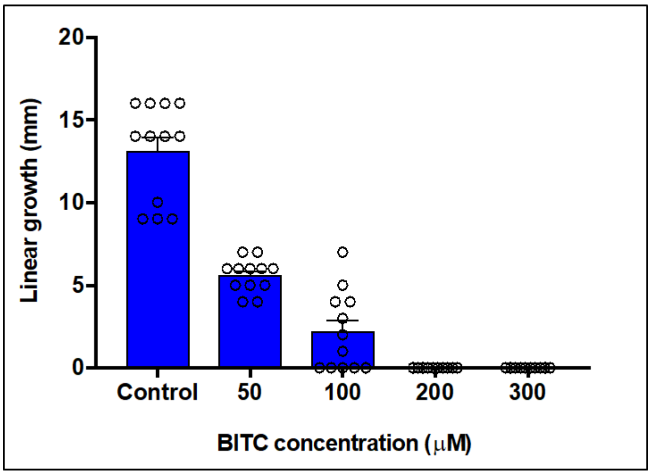

Supplementary Figure 8. Linear growth of *B. cinerea* MDR2 line on BITC. Linear growth of MDR2 isolate on different concentration of BITC as compare to PDA with no BITC (Control) after 24h. Data are displayed as mean values (±SE); n=9. Source data are provided as a Source Data file.

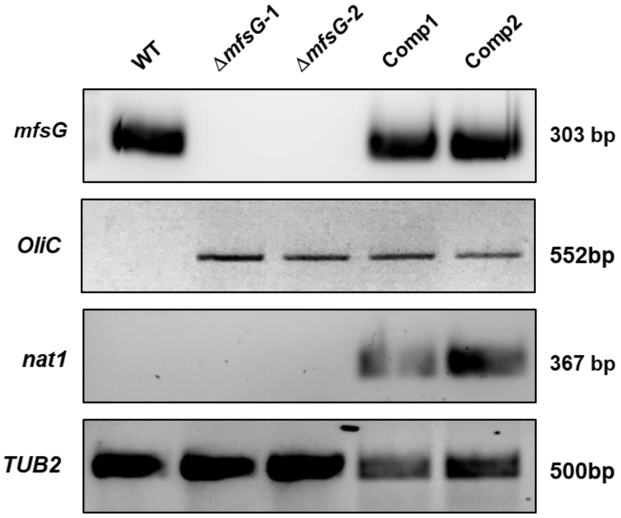

Supplementary Figure 9. Molecular characterization of *B. cinerea mfsG* mutants and complemented mutants. PCR analysis of genomic DNA confirms the absence of *mfsG* in deletion mutants (Δ*mfsG*) and gene restoration in complemented mutants (Comp). The promoter of the hygromycin resistance cassette OliC was present in both mutants and complemented mutants, and *nat1* was detected in the genomic DNA of all putative complemented mutants. Source pictures are provided as a Source Data file.

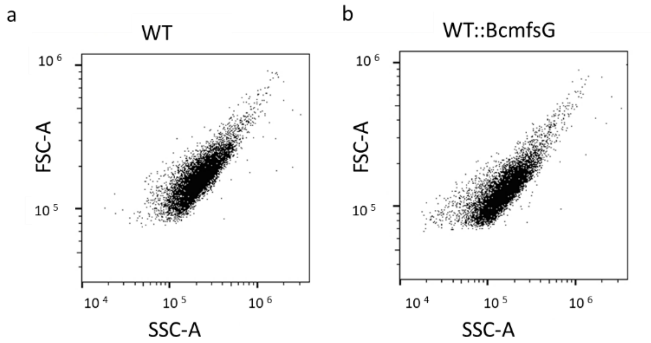

Supplementary Figure 10. Cell distribution in Flow Cytometry analysis. **a** WT cells distribution. **b**. cells expressing BcmfsG (WT::BcmfsG). No sorting or gating were done. 100% of the cells showed normal SSC-A/FSC-A distribution and were included in the analysis. SSC-A, side scatter; FSC-A, forward scatter.

Supplementary Table 1. List of sequences use in phylogenetic analysis.

Isolates	Protein ID	Identity	E-value
Botryotinia fuckeliana B05.10	A0A384JBL8	100.00%	0.00E+00
Botryotinia fuckeliana BcDW1	M7TMD6	94.20%	0.00E+00
Fusarium oxysporum Fo47	W9JDM8	68.80%	0.00E+00
*Fusarium oxysporum f. sp. cubens*e race 4	X0INR7	68.60%	0.00E+00
Alternaria alternata SRC1lrK2f	A0A177DIG1	68.10%	0.00E+00
Podospora anserina FGSC 10383	B2AKJ2	58.00%	1.70E-168
Colletotrichum higginsianum IMI 349063	H1VH25	54.90%	5.70E-170
Leptosphaeria maculans JN3	M1ZJE6	53.10%	2.10E-156
Verticillium alfalfae FGSC 10136	C9SFG4	50.80%	3.90E-141
Fusarium oxysporum f. sp. vasinfectum 25433	X0MNH6	46.20%	1.10E-121
Botryotinia fuckeliana B05.10	A0A384J9T0	45.50%	4.80E-121
Sclerotinia sclerotiorum 1980	A0A1D9QGW2	45.30%	3.80E-119
Aspergillus niger FGSC A1513	A2QYH4	44.50%	1.70E-115
Botryotinia fuckeliana T4	G2XWG5	44.00%	4.60E-116
Botryotinia fuckeliana B05.10	A0A384JBL8	44.00%	4.60E-116
Arthroderma gypseum CBS 118893	E4UST3	43.00%	8.90E-115
Aspergillus flavus AF70	A0A0D9MU50	42.60%	4.00E-117
Botryotinia fuckeliana T4	G2YP26	42.50%	4.20E-115
Botryotinia fuckeliana BcDW1	M7TZC5	42.50%	4.20E-115

Fungal names are in Italic

Supplementary Table 2. Thermodynamic parameters of molecular dockings of BITC to two *mfs-like* from *B. cinerea* B05.10.

	ΔG^0 (Kcal mol^{-1})[a]	Full Fitness (Kcal mol^{-1})[b]
mfs-like 1	-2.48	-1454
mfs-like 2	12.987	-1531

[a] Free Gibbs energy

[b] Score of docking analysis

Supplementary Table 3. Primers used in this study.

Name	Sequence 5´- 3´
attB1hygF	GGGGACAAGTTTGTACAAAAAAGCAGGCTGTTT TCCCAGTCACGACGTT
attB2hygR	GGG GAC CAC TTT GTA CAA GAA AGC TGG GTT GTG GAA TTG TGA GCG GATA
attB3'F	GGG GAC AAC TTT GTA TAG AAA AGT TGG CGG CCG CCC GGG AAA CTC TCA TAT CAC TTG
attB3'R	GGG GAC TGC TTT TTT GTA CAA ACT TGA GGC GTA GCT GTA ATG TGG AAAG
attB5'F	GGG GAC AGC TTT CTT GTA CAA AGT GGG GAT ACC AGT TCA TTC GAC AACAG
attB5'R	GGG GAC AAC TTT GTA TAA TAA AGT TGG CGG CCG CCG AAC CGA AGA ATG TAA TGA CAG C
Hyg F	TGATCGAAAAGTTCGACAGC
Hyg R	CCGGTCGGCATCTACTCTAT
MFSpOliNat1-F	TCTCAACTCCATCACATCACAATCGATCCACACTTGCATCTTACCAATTCTCTAT
MFSpOliNat1-R	AGTGGATCCCGGTCGGCATCTACAATATCTTGCTACCTAAAAGGCTG
Nat1pOli-F	CGCCGGCCGAAACTTTTTTACAACTAGTGATATTGAAGGAGCATTTTT
Nat1pOli-R	TCAAGCTTGCATGCCTGCAGGTAATATTTAAACGCCTACCCTAATTT
MFS-F	ATCGATCACGATCGGATATTGC
MFS-R	CACAAGCCGCTATTCCCATAAC
POliC-F	CCAAACCCTCCGCGTCTTTC
POliC-R	TTGTGACTGGTCGCGAGCTG
BcmfsG-F EcoRI	GGA ATT CAT GCC TTC CAC AGT CAT TCC T
BcmfsG-R xhoI	CCG CTC GAG TTA TAT TTT CGC CCA AAC GTC A
tub2-F	TTCTCGATGTTGTTCGTAAGGAAGC
tub2-R	AGCTTTCGGAGGTCAGAGTTGAGTT
Actin-F	CCCAATCAACCCAAAGTCCAACAG
Actin-R	CAAATCACGACCAGCCATGTC
nat-F	TCACCACCGACACCGTCTTC
nat-R	GGTGCGTTGACGTTGGTGAC

Supplementary References

1. Sievers, F. et al. Fast, scalable generation of high-quality protein multiple sequence alignments using Clustal Omega. *Mol Syst Biol* **7**, 539 (2011).
2. Kumar, S., Stecher, G. & Tamura, K. MEGA7: Molecular Evolutionary Genetics Analysis Version 7.0 for Bigger Datasets. *Mol Biol Evol* **33**, 1870-1874 (2016).
3. Sela, D. et al. Overexpression of AtSHN1/WIN1 Provokes Unique Defense Responses. *PLoS One* **8**, e70146 (2013).
4. Conesa, A. et al. Blast2GO: a universal tool for annotation, visualization and analysis in functional genomics research. *Bioinformatics* **21**, 3674-3676 (2005).
