# Supplementary material for: MFS transporter from Botrytis cinerea provides tolerance to glucosinolate-breakdown products and is required for pathogenicity
Source: Nat Commun. 2019 Jun 28;10:2886. doi: 10.1038/s41467-019-10860-3 (PMC6599007; doi:10.1038/s41467-019-10860-3)
Supplement: Supplementary file 2 — Reporting Summary [file 41467_2019_10860_MOESM2_ESM.pdf]

natureresearch

Corresponding author(s): Maggie Levy

Reporting Summary

Nature Research wishes to improve the reproducibility of the work that we publish. This form provides structure for consistency and transparency in reporting. For further information on Nature Research policies, see Authors & Referees and the Editorial Policy Checklist.

Statistical parameters

When statistical analyses are reported, confirm that the following items are present in the relevant location (e.g. figure legend, table legend, main text, or Methods section).

n/a	Confirmed	
☐	☒	The exact sample size (*n*) for each experimental group/condition, given as a discrete number and unit of measurement
☐	☒	An indication of whether measurements were taken from distinct samples or whether the same sample was measured repeatedly
☐	☒	The statistical test(s) used AND whether they are one- or two-sided<br>*Only common tests should be described solely by name; describe more complex techniques in the Methods section.*
☒	☐	A description of all covariates tested
☐	☒	A description of any assumptions or corrections, such as tests of normality and adjustment for multiple comparisons
☐	☒	A full description of the statistics including central tendency (e.g. means) or other basic estimates (e.g. regression coefficient) AND variation (e.g. standard deviation) or associated estimates of uncertainty (e.g. confidence intervals)
☒	☐	For null hypothesis testing, the test statistic (e.g. *F*, *t*, *r*) with confidence intervals, effect sizes, degrees of freedom and *P* value noted<br>*Give P values as exact values whenever suitable.*
☒	☐	For Bayesian analysis, information on the choice of priors and Markov chain Monte Carlo settings
☒	☐	For hierarchical and complex designs, identification of the appropriate level for tests and full reporting of outcomes
☒	☐	Estimates of effect sizes (e.g. Cohen's *d*, Pearson's *r*), indicating how they were calculated
☐	☒	Clearly defined error bars<br>*State explicitly what error bars represent (e.g. SD, SE, CI)*

Our web collection on statistics for biologists may be useful.

Software and code

Policy information about availability of computer code

Data collection	No software was used
Data analysis	http://wlab.ethz.ch/protter/start/<br>www.swissdock.ch/docking<br>www.sbg.bio.ic.ac.uk/phyre2<br>Clustal Omega<br>MEGA5<br>ASSESS 2.0<br>FlowJo

For manuscripts utilizing custom algorithms or software that are central to the research but not yet described in published literature, software must be made available to editors/reviewers upon request. We strongly encourage code deposition in a community repository (e.g. GitHub). See the Nature Research guidelines for submitting code & software for further information.

Data

Policy information about availability of data

All manuscripts must include a data availability statement. This statement should provide the following information, where applicable:

- Accession codes, unique identifiers, or web links for publicly available datasets
- A list of figures that have associated raw data
- A description of any restrictions on data availability

The datasets generated during and/or analysed during the current study are available from the corresponding author on reasonable request.

Field-specific reporting

Please select the best fit for your research. If you are not sure, read the appropriate sections before making your selection.

☒ Life sciences ☐ Behavioural & social sciences ☐ Ecological, evolutionary & environmental sciences

For a reference copy of the document with all sections, see nature.com/authors/policies/ReportingSummary-flat.pdf

Life sciences study design

All studies must disclose on these points even when the disclosure is negative.

Sample size	fungal linear growth (inhibition) n>3 , lesion size n=17-20, relative florescence Bc n=10, double germ tube n>20<br>qRT-PCR n=3, yeast growth n=3, Flow cytometry n=100000
Data exclusions	no data were exluded
Replication	all attempts at replications were successful
Randomization	plant samples for infection study were taken from at least 10 different plants with random location in the growth room.
Blinding	blind is not relevant since accrued measures of sizes were done automatically using ASSESS 2.0 software

Reporting for specific materials, systems and methods

Materials & experimental systems

n/a	Involved in the study
☐	☒ Unique biological materials
☒	☐ Antibodies
☒	☐ Eukaryotic cell lines
☒	☐ Palaeontology
☐	☒ Animals and other organisms
☒	☐ Human research participants

Methods

n/a	Involved in the study
☒	☐ ChIP-seq
☐	☒ Flow cytometry
☒	☐ MRI-based neuroimaging

Unique biological materials

Policy information about availability of materials

Obtaining unique materials	All unique biological material available from the corresponding author on reasonable request.

Animals and other organisms

Policy information about studies involving animals; ARRIVE guidelines recommended for reporting animal research

Laboratory animals	The phytopathogenic fungi Botrytic cinerea strains B05.10 and yeast cells BY4742 were used
Wild animals	This study did not involve wild animals

Field-collected samples | This study did not involve field collected samples

Flow Cytometry

Plots

Confirm that:

☒ The axis labels state the marker and fluorochrome used (e.g. CD4-FITC).

☒ The axis scales are clearly visible. Include numbers along axes only for bottom left plot of group (a 'group' is an analysis of identical markers).

☒ All plots are contour plots with outliers or pseudocolor plots.

☒ A numerical value for number of cells or percentage (with statistics) is provided.

Methodology

Sample preparation	Yeast were grown in YPD with galactose uder constant agigation of 150 rpm for 5-8 h to OD595 of 0.5. FITC was then added (10ug/ml) with 50mM sodium citrate and cells were incubated at 37oC for 30 min. Cells where then washed 3 times with YND galactose and incubate at 30oC for 2h before subjected to flow cytometry.
Instrument	CytoFlex Beckman
Software	Cytoflex and flowJo
Cell population abundance	No sorting or gating were made. 100% of the cells showed normal SSC-A/FSC-A distribution and were included in the anlysis
Gating strategy	no gating was done 100% of the cells were used for analysis.

☒ Tick this box to confirm that a figure exemplifying the gating strategy is provided in the Supplementary Information.
